# Sedation and Pain Management in Neonates Undergoing Therapeutic Hypothermia for Hypoxic-Ischemic Encephalopathy

**DOI:** 10.3390/children12020253

**Published:** 2025-02-19

**Authors:** Artemiy Kokhanov, Peggy Chen

**Affiliations:** 1 Phoenix Children’s, Phoenix, AZ 85016, USA; 2 MemorialCare Miller Women’s and Children’s Hospital Long Beach, Long Beach, CA 90806, USA

**Keywords:** hypoxic-ischemic encephalopathy, therapeutic hypothermia, sedation, dexmedetomidine, opioids, benzodiazepines, anticonvulsants

## Abstract

Hypoxic-ischemic encephalopathy (HIE) is a common cause of significant neonatal morbidity and mortality. The stronghold of the treatment for moderate-to-severe HIE is therapeutic hypothermia (TH) which provides a neuroprotective effect. However, it also is associated with pain and stress. Moreover, neonates with HIE are subjected to a significant number of painful procedures. Untreated pain during the early neonatal period may entail future challenges such as impaired brain growth and development as well as impaired pain sensitivity later in life. Hereby, the provision of adequate sedation and alleviation of pain and discomfort is essential. There are currently no universally accepted guidelines for sedation and pain management for this patient population. In this review, we highlight non-pharmacologic and pharmacologic methods currently in use to provide comfort and sedation to patients with HIE undergoing TH.

## 1. Introduction

Hypoxic-ischemic encephalopathy (HIE) is a common cause of significant neonatal morbidity and mortality [1]. In developed countries, the incidence of HIE varies between one and three in one thousand live births [2,3]. In developing countries, the incidence of neonatal HIE is estimated to be 10 times higher [1,4]. Globally, HIE accounts for 6.1 million years lived with disability and 50 million disability-adjusted life years [5]. The mainstay of the treatment for moderate to severe encephalopathy is therapeutic hypothermia (TH) initiated within 6 h of birth [6,7]. During TH, the core body temperature of the affected neonate is lowered to 33.5 degrees Celsius and then maintained for 72 h, followed by slow rewarming until the body temperature returns back to normal [8].

TH provides neuroprotective effects and reduces death and disability in asphyxiated neonates [8,9]. At the same time, TH is associated with pain and stress [10,11,12]. The physiologic homeostasis of the human body requires a stable body temperature [13]. When the body temperature is lowered during TH, the homeostasis is altered [14]. Various offset responses follow, such as brown adipose tissue thermogenesis, agitation, restlessness, and crying as well as the activation of hypothalamic–pituitary–adrenal axis and release of cortisol [15,16,17]. Furthermore, neonates who are treated with TH are often subjected to a greater number of painful procedures than other patients requiring neonatal intensive care [11]. Unaddressed pain during the early newborn period may lead to future problems such as impaired brain growth and development as well as impaired pain sensitivity later in life [18,19,20]. Therefore, the alleviation of pain, discomfort, and stress response during TH is necessary. Unfortunately, at this time there are no universally accepted guidelines for sedation and pain management for this patient population [21]. In this article, we review methods and strategies to provide comfort and sedation to neonates undergoing TH.

## 2. Non-Pharmacologic Ways to Provide Comfort

Up to now, non-pharmacologic interventions to alleviate discomfort during TH have not been systematically studied [22]. However, neuroprotective developmentally supportive care comprising a range of strategies that aim to reduce the influence of stressors on the developing brain must be considered. These should be considered as an adjunct to pharmacologic sedation and be used alongside it. Facilitated tucking (a practice of containing the newborn by placing the caregiver’s hands on both head and lower extremities to maintain a ‘folded-in’ position) along with non-nutritive sucking are simple and effective strategies to reduce procedural pain [23]. A Cochrane review by Riddell showed that facilitated tucking and non-nutritive sucking reduce pain reactivity and improve immediate pain regulation; however, the certainty of the evidence was low [23].

The odor of maternal breast milk (but not that of donor milk or formula) has also been shown to reduce infant pain responses. A study by Nishitani et al. showed decreased behavioral indices of the pain response (grimacing, crying, and motor activity) after the patients were presented with the scent of their own mother’s milk. In addition, the increase in a biochemical index of pain response (by measuring cortisol levels in the infant’s saliva) was inhibited. The breast milk odors alleviated pain responses in their own newborns, but not other women’s newborns, indicating that the calming action of breast milk odors is limited to a woman’s own child [24]. Other earlier studies showed similar results [25,26]. However, to our knowledge, there are no studies evaluating olfaction in sedated neonates. It is unknown to what extent it is sustained.

The practice of placing neonates on nil per os (NPO) status during TH has been predominant historically. However, more recent data have shown that the provision of enteral nutrition is not only safe but also beneficial during TH. A study by Alburaki et al. from 2022 concluded that feeding while on therapeutic hypothermia was safe and also was associated with a significant reduction in time needed to reach full-volume enteral feeds, as well as a greater rate of feeding of breast milk at discharge, compared to keeping patients NPO during TH [27]. A retrospective study from the United Kingdom conducted in 2021 showed that necrotizing enterocolitis occurs rarely in term and near-term neonates undergoing TH, and in fact, was less common in those patients who were enterally fed. The authors also found that early introduction of enteral feeds was associated with beneficial outcomes (e.g., a higher proportion of patients breastfeeding at discharge, shorter length of hospital stay); however, residual confounding could not be entirely ruled out [28].

Other than providing comfort, feeding provides various other benefits, such as improving the structural and functional integrity of the gut, decreasing systemic inflammatory responses, and augmenting gut microbiome diversity [29]. As a result, the practice of providing enteral nutrition has gained acceptance by many neonatal intensive care units [29].

It is also possible to safely involve parents in various aspects of care during TH [30]. Every family must be carefully directed to participate in their neonate’s care gradually. Although kangaroo care is not feasible, parental holding while on the cooling blanket has been shown to be a safe practice that does not lead to clinical instability. In a small sample study by Craig et al., parental holding was formally assessed, and the results supported its feasibility [31]. In this study, parental holding did not seem to impact the effect of therapeutic hypothermia. The mean body temperature prior to holding was 33.4 °C and 33.5 °C before placing the patient back on the bed. Larger studies are needed to further assess the impact of parental holding on neonates undergoing TH.

Consideration of non-pharmacologic pain and stress alleviation techniques should be very cautiously considered in neonates with severe brain damage and low Apgar scores, although reviews and primary studies do exist. The results of these studies should be taken into consideration with caution.

## 3. Pharmacologic Sedation

### 3.1. Overview

Pharmacologic sedation and analgesia can be used with the goal of alleviating perceived pain or discomfort from therapeutic hypothermia. However, in practice, the use and management of pharmacologic sedation are highly variable. This is likely due to several different factors. The literature regarding long-term neurologic outcomes of neonatal intensive care unit (NICU) survivors is not straightforward. On the one hand, there is substantial evidence in normothermic neonates showing that pain-related stress can be harmful to the developing brain and is associated with a smaller cerebral cortex, cerebellum, and subcortical structures at school age [17,32,33], particularly in preterm infants. However, other studies have shown that neonatal exposure to medications like morphine, despite leading to decreased signs of pain, is associated with smaller cerebellar size in preterm infants at term-corrected age, poorer motor and cognitive outcome at 18 months of age, and increased risk for intraventricular hemorrhage (IVH) and periventricular leukomalacia (PVL) [34,35]. In addition, most initial therapeutic hypothermia clinical trials did not have specific sedation protocols to help guide future practice [36,37,38,39], with only one trial, the neo.nEURO.network trial, specifying pharmacologic sedation [40]. Finally, preclinical models show conflicting results regarding the impact of sedation on neurologic outcomes post therapeutic hypothermia. A study by Thoresen et al. showed that in the absence of sedation, 24 h of therapeutic hypothermia was no longer neuroprotective in piglets [17]; however, a sheep model showed that the benefits of therapeutic hypothermia persisted without sedation [41]. In addition, other animal model studies suggest that exposure to sedative–analgesics has long-term deleterious effects on brain development [42,43].

While there are neonatal pharmacokinetic and long-term outcome data on several sedative–analgesic medications, there is a paucity of data in infants who are undergoing therapeutic hypothermia, which is important given the added complication of the changes in biochemical and physiologic processes that can occur when the body temperature deviates from the temperatures which optimize physiologic homeostasis. With hypothermia, the body undergoes vasoconstriction, bradycardia, a decrease in cardiac output by 7% for every degree Celsius below 37, and a decrease in metabolic rate by 5–8% for every degree Celsius below 37. In addition, with reduced CO_2_ production from decreased metabolism and higher CO_2_ dissolution in blood, there is a need to be cautious in considering cerebral blood flow, which may already be affected by decreased cardiac output [22]. In addition, these changes are also thought to lead to significant changes in pharmacokinetics, making dosing of medications an additional confounding factor on top of choosing which medication or using medication at all.

There are several classes of medications that are generally favored when choosing a sedative–analgesic in neonates undergoing therapeutic hypothermia, including opioids, alpha-2 adrenergic receptor agonists, and benzodiazepines. Also, anti-excitatory medications are occasionally used, not in their role as an antiseizure medication but rather for sedation.

Below we review different classes of pharmacologic agents, not in any particular order of preference.

### 3.2. Opioids

Opioids are among the most used medications in the NICU both historically and currently. One of the therapeutic hypothermia randomized controlled trials (RCTs)—the neo.nEURO.network trial—specified the use of opioids for discomfort [40]. Two relatively recent surveys published on care provided during therapeutic hypothermia both showed that opioids were the most common pharmacologic agents used to provide sedation and/or analgesia for neonates with HIE. One study found that pre-emptive opioid infusions were standard care for ventilated neonates in 86% of responding centers in the United Kingdom and 50% of responding centers in the United States and Canada. Opioids remained commonly used in non-ventilated neonates, with 40% of responding centers in the United Kingdom and 44% of centers in the United States and Canada standardly using pre-emptive opioid infusions [44]. The other study received survey responses from 24 out of the 26 total tertiary care NICUs in Canada and found that 54% of the centers routinely provided sedation during therapeutic hypothermia and that morphine was the most frequently used (66%), followed by fentanyl (17%) [45]. In addition, a retrospective chart review study of 125 NICUs across the United States also showed that most neonates received at least one opioid (64%), with morphine (33%) and fentanyl (36%) having approximately equal usage [46]. They also found that compared to neonates who did not receive any sedation or only received non-opioids (i.e., benzodiazepines), neonates who received opioids had a greater median duration of mechanical ventilation.

Given the prevalence of opioid use, particularly in this population that is already neurologically vulnerable due to HIE, the neurologic outcomes of neonates with HIE who undergo therapeutic hypothermia and are exposed to opioids are of significant interest. A secondary analysis of the National Institute of Child Health and Human Development (NICHD) Neonatal Research Network (NRN) therapeutic hypothermia RCTs showed that at 18 months, there was no association between exposure to drugs classified as sedative–analgesics (which included morphine and fentanyl) and death and disability among survivors [47]. The authors, however, did note a significant caveat that there were no defined protocols for sedation-analgesia within the trial. On the other hand, another secondary analysis study showed opposing results. A secondary analysis of the Magnetic Resonance Biomarkers in Neonatal Encephalopathy (MARBLE) study found that pre-emptive morphine did not provide any neuroprotective benefits and was associated with increased adverse neurodevelopmental outcomes when adjusted for the severity of encephalopathy as well as longer hospital stays [48].

Another study looked at cumulative doses of morphine and fentanyl on neurologic outcomes at 18–24 months of age based on Bayley-3 neurodevelopmental assessments of cognition, language, and motor function [49]. The authors stratified their results based on early (<6 h of life) amplitude-integrated electroencephalography (aEEG) patterns to separate infants into mild, moderate, and severe encephalopathy subgroups and did not find a significant association between cumulative sedation-analgesia dose and Bayley-3 scores in any of the assessment domains. They also found that the duration of sedation-analgesia was highly correlated with the duration of mechanical ventilation, again highlighting the prevalence of respiratory depression with the use of opiates.

The effect of cumulative fentanyl dosage on neurologic outcomes was published by Lugli and colleagues in 2022 [50]. Severe neurodevelopmental impairment in this study was defined by the diagnosis of cerebral palsy, Griffith’s development quotient < 70, major sensorineural deficit, or severe brain lesion (extensive involvement of cortex and/or basal ganglia). The authors did not find a significant negative impact of fentanyl use during therapeutic hypothermia or an association between higher cumulative fentanyl dosage with poorer neurologic outcomes. Again, however, they found that respiratory depression was an associated adverse effect.

Given the variation in practice and frequency of adverse effects associated with opioid use in therapeutic hypothermia, pharmacokinetic studies can provide substantial information and guidance for practice. Published pharmacokinetic studies on morphine found that clearance in neonates with HIE undergoing therapeutic hypothermia was approximately 25–50% lower compared to their normothermic counterparts, and based on this, recommendations were made for a loading dose of 0.05 mg/kg followed by a continuous infusion starting at 0.005 mg/kg/h to maintain desired morphine plasma concentrations between 10–40 ng/mL [51,52]. A phase 1 clinical trial of continuous fentanyl infusion in neonates with HIE undergoing therapeutic hypothermia was launched in 2023 by Lugli and colleagues and will hopefully be able to provide valuable pharmacokinetic information for similar guidance on fentanyl dosage in the future [53].

### 3.3. Alpha-2 Adrenergic Agonists

One class of medications that has been gaining increasing popularity over the past several years are alpha-2 adrenergic receptor agonists, specifically dexmedetomidine and clonidine. Central alpha-2 agonists have several characteristics that may make them beneficial during therapeutic hypothermia. First, alpha-2 receptor agonists modulate thermoregulatory parameters for shivering and sweating by blocking presynaptic alpha-2 receptors in the brainstem and reducing norepinephrine release from sympathetic ganglia involved in shivering and non-shivering thermogenesis [54,55]; they have also been shown to be more effective in reducing post-operative shivering than opioids [56]. In addition, alpha-2 adrenergic receptor agonists provide sedation and mild analgesia while avoiding many of the undesirable adverse effects of opioids, including respiratory depression, hypotension, and gastrointestinal dysmotility [55,57,58,59,60,61,62,63]. Finally, there is evidence in animal models [61,64,65,66,67] and adult clinical studies [68] that alpha-2 receptor agonists may be neuroprotective after hypoxic-ischemic insult.

Clonidine is an alpha-2 adrenergic receptor agonist that was initially used primarily as an antihypertensive agent but has been successfully used for its sedating and analgesic effects in the neonatal population in treating neonatal abstinence syndrome [63]. A study published by Gauda et al. in 2022 looked at the use of IV clonidine for sedation in neonates during therapeutic hypothermia, first starting with a dose of 1 mcg/kg q6h, which was determined based on the dose used in neonatal abstinence syndrome (NAS) of 1 mcg/kg q4h but reduced by 30% based on studies that showed 25–40% decreased clearance of aminoglycosides during therapeutic hypothermia [55]. Due to two out of the initial four infants in the trial requiring spacing of dosing due to bradycardia or hypotension, initial dosing was spaced to q8h for the remainder of the study. The results of this study found that infants treated with intravenous clonidine 1 mcg/kg q8h given over 30 min during the cooling period of therapeutic hypothermia did not have significant bradycardia or hypotension but did have significantly decreased need for opioid use and increased time spent within the optimal core body temperature range compared to the original control group. Pharmacokinetic data collected during the study showed a decrease in clearance of 21.8% and a decrease in volume of distribution by 28.4% of clonidine compared to normothermic term infants with NAS. The authors concluded that clonidine could be safely used in neonates with HIE being treated with therapeutic hypothermia and may help reduce the use and adverse effects of higher-dose opiate use.

More research has been directed towards the investigation of dexmedetomidine. Dexmedetomidine is a highly lipophilic and selective alpha-2 adrenergic receptor agonist, which is metabolized in the liver and excreted by the kidneys. Due to the aforementioned benefits, several studies have been conducted recently looking at the use of dexmedetomidine in neonatal encephalopathy undergoing therapeutic hypothermia. While previous studies looking at safety, efficacy, and pharmacokinetics have shown that dexmedetomidine was effective and well-tolerated for sedation in preterm and full-term neonates [69], studies specifically looking at its use in hypothermia did not come until later. Particular concerns were noted in the neonatal population with HIE undergoing therapeutic hypothermia given the change in pharmacokinetics that can be seen with therapeutic hypothermia, as well as the combination of the known hemodynamic adverse effects of alpha-2 receptor agonists (bradycardia, hypotension) in the context of already reduced heart rate and cardiac output in neonates with HIE undergoing therapeutic hypothermia. Several recent studies have attempted to address these concerns.

A retrospective study by Elliott and colleagues specifically looking at the effect of dexmedetomidine on heart rate was published in 2022, with results showing that at doses of 0.2–1 mcg/kg/h, mean heart rate and nadir were overall lower in neonates treated with dexmedetomidine compared to those treated with fentanyl, but that adequate perfusion was maintained [70]. The authors concluded that while there was an association with lower heart rate, given the lack of impairment of perfusion and potential neuroprotective effects, dexmedetomidine could be successfully used in neonates with HIE undergoing therapeutic hypothermia and noted that their center continues to use it as first-line therapy during therapeutic hypothermia. A more recent retrospective cohort study looking at the safety and efficacy of dexmedetomidine monotherapy during therapeutic hypothermia also found that bradycardia was the most common adverse effect but that associated hypotension requiring treatment was very rare [71]. The starting dose used in this study was 0.2 mcg/kg/h with titration by 0.1 mcg/kg/h as needed to achieve adequate sedation or in response to hemodynamic (heart rate) changes with a median maximum dose of 0.3 mcg/kg/h. Based on their results, the authors again reached similar conclusions and recommendations to Elliott and colleagues.

However, other studies have not found the same significant change in hemodynamic parameters. A single-center retrospective study published by O’Mara and Weiss in 2018 showed that the use of dexmedetomidine both as an adjunct to fentanyl and as monotherapy was generally safe and led to substantially reduced fentanyl usage, reduced time to full feeds, and reduced central line days [57]. There were no negative effects on hemodynamic parameters or need for mechanical ventilation. The conclusion was that dexmedetomidine was safe and effective for sedation in neonates undergoing therapeutic hypothermia, especially non-intubated infants. Another study comparing dexmedetomidine to intermittent morphine also showed that dexmedetomidine was safe and effective for sedation and analgesia during therapeutic hypothermia, with no significant differences in pain scores, heart rate, mean arterial pressure, SpO2, vasopressor use, video-electroencephalography (vEEG) patterns, or magnetic resonance imaging (MRI) NICHD scores post therapeutic hypothermia between groups. While they did not find a significant reduction in the duration of invasive mechanical ventilation, they noted that all the patients treated with dexmedetomidine were able to wean from ventilator support while some patients on morphine required an increase in ventilator support [72].

Two other studies have shown a decrease in invasive mechanical ventilation required in infants who were treated with dexmedetomidine compared to opioids. A retrospective study comparing dexmedetomidine to fentanyl in neonates with HIE undergoing therapeutic hypothermia found that infants who were treated with dexmedetomidine had significantly decreased time to extubation [73]. The authors also found that infants in the dexmedetomidine group had a significantly shorter average time to discontinuation of sedation after rewarming and an earlier average time to full enteral feedings compared to infants who received fentanyl. They also found that the group treated with dexmedetomidine had significantly decreased needs for bolus doses of sedatives during therapeutic hypothermia without a difference in the number of uncontrolled agitation scores or the need for additional scheduled sedation medication. Separately, a single-center prospective randomized study compared dexmedetomidine infusion to a group of control medications of morphine, sodium oxybutyrate, or diazepam at standard recommended doses in term infants with moderate-to-severe HIE being treated with therapeutic hypothermia [74]. Using biochemical and cerebral Doppler information obtained on days 1 and 3, the author established similar severity of hypoxic-ischemic insult and preservation of cerebral blood flow in both groups. In this study, the infants in the dexmedetomidine group were significantly more likely to be extubated by day 7 of life compared to those in the control group. Several other results supporting the efficacy of dexmedetomidine were also found by the author. There was no significant difference in hypotension or bradycardia found; in fact, the mean arterial pressure was higher in the group receiving dexmedetomidine compared to the control. Cerebral near-infrared spectroscopy (NIRS) measurements were compared between groups with a lower average in the dexmedetomidine group compared to the control group; however, this did not reach statistical significance, and both groups overall remained within the targeted reference range throughout treatment. In addition, the author found significantly lower rates of seizures during the first day of treatment in the dexmedetomidine group compared to the control group, as well as a 7-fold lower rate of cerebral leukomalacia in the dexmedetomidine group compared to the control group.

In 2020, a phase 1 study was published on the pharmacokinetics of dexmedetomidine specifically in neonates with HIE undergoing therapeutic hypothermia [75]. The study protocol started infants on a dose of 0.2 mcg/kg/h and gradually increased to 0.4 mcg/kg/h before discontinuing without weaning 6 h post-rewarming. The authors found several significant differences between their study population and infants without HIE not undergoing therapeutic hypothermia. First, they found that plasma concentrations after initiation at these doses were very low in the first few hours before gradually increasing and plateauing 12–24 h post-infusion, which was delayed compared to normothermic infants without HIE. They also found an increased time to achieve steady state change after a change in dose of 28 h compared to 13 h in normothermic infants without HIE. Plasma concentrations declined exponentially and were detectable for up to 43 h after discontinuation of infusion with an elimination half-life of approximately 7.3 h, longer than the estimated value of 3 h for normothermic infants without HIE. They also found minimal recovery in urine or feces of unchanged drug, suggesting that renal function was not as important in clearance but postulated that depressed hepatic function along with a larger volume of distribution and shorter mean residence time could all be contributors to lower clearance compared to non-HIE normothermic infants. Based on their study results, the authors concluded that dexmedetomidine seemed safe for use in infants with HIE undergoing therapeutic hypothermia but may benefit from more rapid escalation and/or a loading dose to achieve effective levels in a timely manner. The potential benefit of a loading dose was also discussed by Acun and colleagues in their 2024 study, given their finding that the majority of infants treated with dexmedetomidine who required at least one bolus dose of opioid or benzodiazepine to achieve appropriate sedation did so within the first 24 h of starting dexmedetomidine [71].

Finally, in 2021, the Dexmedetomidine Use in Infants Undergoing Cooling due to Neonatal Encephalopathy (DICE) Trial, a phase II multicenter randomized safety and pharmacokinetics study, was launched [76]. The results of this trial will hopefully give insight into questions of safety, pharmacokinetics, and neurodevelopmental outcomes regarding the use of dexmedetomidine vs. morphine in this population.

### 3.4. Benzodiazepines

Benzodiazepines have also been used for sedation in the NICU setting, including during therapeutic hypothermia. The aforementioned retrospective study of 125 NICUs across the United States by Berube and colleagues found that benzodiazepines were the second most common drug class used for sedation during therapeutic hypothermia, with 49% of all neonates in the study receiving at least 1 dose [46]. Of those who received a benzodiazepine, the most frequently used benzodiazepine was midazolam at 35%, followed by lorazepam at 17%. The authors also noted that for neonates who received benzodiazepine monotherapy, the median duration of ventilator days was 2 days compared to 4 days for those who received opioid monotherapy.

Midazolam specifically was studied for use in neonates with HIE undergoing therapeutic hypothermia, both as a sedative as well as for anticonvulsive treatment refractory to phenobarbital [77]. The sedative dose was lower and without a loading dose, while the anticonvulsive regimen consisted of a loading dose followed by continuous infusion and further escalation. The authors found that hypotension was a significant adverse effect of midazolam, with 64% of neonates experiencing at least one hypotensive event during treatment despite inotropic support. Of those who received midazolam for control of seizures refractory to phenobarbital, only 23% achieved sufficient control on a maximum dose, with the remaining requiring additional anticonvulsants. The authors concluded that given the hemodynamic concerns and limited efficacy for refractory seizures, midazolam was a less suitable medication for use in therapeutic hypothermia. Another article by McPherson et al. reviewing the use and indications for sedation in neonates with HIE undergoing therapeutic hypothermia also cautioned against the routine use of benzodiazepines, given the risk of impacting hemodynamic stability as well as concerns regarding the effect on neurodevelopment considering the substantial role that gamma-aminobutyric acid (GABA)-mediated activity plays in neuronal and synaptic development [22].

### 3.5. Other Anticonvulsants

Seizures are frequently seen in neonates with HIE undergoing therapeutic hypothermia. and the use of other (non-benzodiazepine) anticonvulsant medications seems to overall be limited to the treatment of seizures. The aforementioned survey of Canadian tertiary NICUs found that phenobarbital was by far the most frequent choice of anti-epileptic, with 92% of NICUs using it as their first-line treatment [45]. The same survey found that for second-line treatments, phenytoin was the most frequent (42%) followed by levetiracetam (5%). Phenobarbital is also the first-line treatment for neonatal seizures in Europe and the initial treatment for seizures in the previously mentioned study on midazolam [77].

The secondary analysis study of the NICHD NRN therapeutic hypothermia RCTs found an independent association with anticonvulsive receipt (with or without sedative–analgesics) and increased risk of disability at 18 months among survivors (37%), and death or disability (58%). By comparison, infants who received sedative–analgesic use alone had 13% and 32% incidence, respectively, and infants who had no exposure to pharmaceutical sedation had 14% and 34% incidence, respectively [47]. However, it is difficult to interpret these findings, as the classification of medications, as either sedative–analgesics or anticonvulsants, had overlap with phenobarbital in both categories, with midazolam categorized as a sedative–analgesic, and lorazepam classified as an anticonvulsant.

There have also been preclinical studies in rodent models showing the potential benefit of anticonvulsant medications, particularly as an adjunctive to extend the treatment window for the initiation of therapeutic hypothermia. In a neonatal rat stroke model, the combination of early topiramate and delayed hypothermia of 3 h was found to have improved functional outcomes and reduction in brain infarction 8 days after recovery [78]. These benefits were not seen in rats treated only with either topiramate or delayed hypothermia alone. Another study looking at early phenobarbital administration and delayed hypothermia in a neonatal rat model of hypoxic-ischemic encephalopathy also showed similar findings [79]. While these studies show promising results, significant further research is needed in this area before the application and significance to the human neonatal population can be determined. Besides that, however, antiepileptic medications such as phenobarbital, phenytoin, diazepam, and clonazepam among others have been found to cause apoptotic degeneration in rat models of developing brain [80]. This effect needs to be considered when choosing to use these agents.

## 4. Conclusions

Defining appropriate strategies for sedation in newborns being treated with therapeutic hypothermia is of the ultimate importance. Management of these neonates frequently requires an abundance of stress-inducing and painful procedures. Inadequate management of neonatal pain might result in negative long-term outcomes. The use of non-pharmacologic approaches to alleviate pain should be maximized. Sedation and pain management using pharmacologic agents should be optimized to achieve the optimal level of patient comfort (Table 1). It is important to take into consideration the individual clinical presentation of each patient when choosing the right sedative–analgesic medication since they may have different benefits and side effect profiles. More studies are needed to assess the safety and efficacy as well as the effect on long-term neurodevelopmental outcomes of different sedation regimens. Similarly, it would be helpful to know the effects of various sedative agents on electroencephalography as well as their effects on neonatal hemodynamics.

## Figures and Tables

**Table 1:** Sedation and pain management during therapeutic hypothermia.

Non-Pharmacologic	Pharmacologic
Forms the foundation for providing comfort for neonates on therapeutic hypothermia	Most trials did not use any sedation alongside therapeutic hypothermia.
All newborns should receive developmentally appropriate strategies during therapeutic hypothermia and painful procedures	Neonatal pain assessment and sedation scale (N-PASS) as sedation evaluation has not been validated in this population. Use clinical judgment to keep the infant comfortable.
Non-pharmacologic strategies should be used in addition to pharmacologic strategies.	Medication choices:
Facilitated tucking Positioning and containment Reduction in stimuli (light and sound) Non-nutritive sucking Oral care with maternal breast milk Provision of small-amount enteral feeds	Opioids (e.g., morphine, fentanyl)—have the strongest basis in evidence Alpha-2 adrenergic agonists (e.g., dexmedetomidine, clonidine)—may be considered as an alternative to opioids in a setting of appropriate monitoring for bradycardia Benzodiazepines (e.g., lorazepam, midazolam)—should not be routinely used due to the high risk of hemodynamic instability and the potential to increase aberrant neuronal and synaptic development

## Abbreviations

The following abbreviations are used in this manuscript:

HIE	hypoxic-ischemic encephalopathy
TH	therapeutic hypothermia
NPO	nil per os
NICU	neonatal intensive care unit
IVH	intraventricular hemorrhage
PVL	periventricular leukomalacia
RCT	randomized controlled trial
NICHD	National Institute of Child Health and Human Development
NRN	Neonatal Research Network
MARBLE	Magnetic Resonance Biomarkers in Neonatal Encephalopathy
aEEG	amplitude-integrated electroencephalography
NAS	neonatal abstinence syndrome
vEEG	video-electroencephalography
MRI	magnetic resonance imaging
DICE	Dexmedetomidine Use in Infants Undergoing Cooling Due to Neonatal Encephalopathy
GABA	gamma-aminobutyric acid

## References

[1] Acun C., Karnati S., Padiyar S., Puthuraya S., Aly H., Mohamed M. (2022). Trends of neonatal hypoxic-ischemic encephalopathy prevalence and associated risk factors in the United States, 2010 to 2018. Am. J. Obstet Gynecol..

[2] Shipley L., Gale C., Sharkey D. (2021). Trends in the incidence and management of hypoxic-ischaemic encephalopathy in the therapeutic hypothermia era: A national population study. Arch. Dis. Child. Fetal Neonatal Ed..

[3] Vega-Del-Val C., Arnaez J., Caserío S., Gutierrez E.P., Benito M., Castanon L., Garcia-Alix A., IC-HIE Study Group (2021). Temporal trends in the severity and mortality of neonatal hypoxic-ischemic encephalopathy in the era of hypothermia. Neonatology.

[4] Ezenwa B.N., Olorunfemi G., Fajolu I., Adeniyi T., Oleolo-Ayodeji K., Kene-Udemezue B., Olamijulo J.A., Ezeaka C. (2021). Trends and predictors of in-hospital mortality among babies with hypoxic ischaemic encephalopathy at a tertiary hospital in Nigeria: A retrospective cohort study. PLoS ONE.

[5] Lee A.C.C., Kozuki N., Blencowe H., Vos T., Bahalim A., Darmstadt G.L., Niermeyer S., Ellis M., Robertson N.J., Cousens S. (2013). Intrapartum-related neonatal encephalopathy incidence and impairment at regional and global levels for 2010 with trends from 1990. Pediatr. Res..

[6] Jacobs S.E., Berg M., Hunt R., Tarnow-Mordi W.O., Inder T.E., Davis P.G. (2013). Cooling for newborns with hypoxic ischaemic encephalopathy. Cochrane Database Syst. Rev..

[7] Chiang M.C., Jong Y.J., Lin C.H. (2017). Therapeutic hypothermia for neonates with hypoxic ischemic encephalopathy. Pediatr. Neonatol..

[8] Wassink G., Davidson J.O., Dhillon S.K., Zhou K., Bennet L., Thoresen M., Gunn A.J. (2019). Therapeutic hypothermia in neonatal hypoxic-ischemic encephalopathy. Curr. Neurol. Neurosci. Rep..

[9] Kurisu K., Kim J.Y., You J., Yenari M.A. (2019). Therapeutic hypothermia and neuroprotection in acute neurological disease. Curr. Med. Chem..

[10] Hoffman K., Bromster T., Hakansson S., van den Berg J. (2013). Monitoring of pain and stress in an infant with asphyxia during induced hypothermia: A case report. Adv. Neonatal Care.

[11] Axelin A., Cilio M.R., Asunis M., Peloquin S., Franck L.S. (2013). Sleep-wake cycling in a neonate admitted to the NICU: A video-EEG case study during hypothermia treatment. J. Perinat. Neonatal Nurs..

[12] Üner I.L., Johansen T., Dahle J., Persson M., Stiris T., Andresen J.H. (2019). Therapeutic hypothermia and N-PASS; results from implementation in a level 3 NICU. Early Hum. Dev..

[13] Tansey E.A., Johnson C.D. (2015). Recent advances in thermoregulation. Adv. Physiol. Educ..

[14] Wood T., Thoresen M. (2015). Physiological responses to hypothermia. Semin. Fetal Neonatal Med..

[15] Astrup A. (1986). Thermogenesis in human brown adipose tissue and skeletal muscle induced by sympathomimetic stimulation. Acta Endocrinol. Suppl..

[16] van Marken Lichtenbelt W.D., Schrauwen P. (2011). Implications of nonshivering thermogenesis for energy balance regulation in humans. Am. J. Physiol. Regul. Integr. Comp. Physiol..

[17] Thoresen M., Satas S., Løberg E.M., Whitelaw A., Acolet D., Lindgren C., Penrice J., Robertson N., Haug E., Steen P.A. (2001). Twenty-four hours of mild hypothermia in unsedated newborn pigs starting after a severe global hypoxic-ischemic insult is not neuroprotective. Pediatr. Res..

[18] Walker S.M. (2017). Translational studies identify long-term impact of prior neonatal pain experience. Pain.

[19] Williams M.D., Lascelles B.D.X. (2020). Early neonatal pain—A review of clinical and experimental implications on painful conditions later in life. Front. Pediatr..

[20] Walker S.M. (2019). Long-term effects of neonatal pain. Semin. Fetal Neonatal Med..

[21] Joshi M., Muneer J., Mbuagbaw L., Goswami I. (2023). Analgesia and sedation strategies in neonates undergoing whole-body therapeutic hypothermia: A scoping review. PLoS ONE.

[22] McPherson C., Frymoyer A., Ortinau C.M., Miller S.P., Groenendaal F., Newborn Brain Society Guidelines and Publications Committee (2021). Management of comfort and sedation in neonates with neonatal encephalopathy treated with therapeutic hypothermia. Semin. Fetal Neonatal Med..

[23] Pillai Riddell R.R., Bucsea O., Shiff I., Chow C., Gennis H.G., Badovinac S., DiLorenzo-Klas M., Racine N.M., Kohut S.A., Lisi D. (2023). Non-pharmacological management of infant and young child procedural pain. Cochrane Database Syst. Rev..

[24] Nishitani S., Miyamura T., Tagawa M., Sumi M., Takase R., Doi H., Moriuchi H., Shinohara K. (2009). The calming effect of a maternal breast milk odor on the human newborn infant. Neurosci. Res..

[25] Mellier D., Bezard S., Caston J. (1997). Études exploratoires des relations intersensorielles olfaction-douleur. Enfance.

[26] Rattaz C., Goubet N., Bullinger A. (2005). The calming effect of a familiar odor on full-term newborns. J. Dev. Behav. Pediatr..

[27] Alburaki W., Scringer-Wilkes M., Dawoud F., Oliver N., Lind J., Zein H., Leijser L.M., Esser M.J., Mohammad K. (2022). Feeding during therapeutic hypothermia is safe and may improve outcomes in newborns with perinatal asphyxia. J. Matern. Fetal Neonatal Med..

[28] Gale C., Longford N.T., Jeyakumaran D., Ougham K., Battersby C., Ojha S., Dorling J. (2021). Feeding during neonatal therapeutic hypothermia, assessed using routinely collected National Neonatal Research Database data: A retrospective, UK population-based cohort study. Lancet Child. Adolesc. Health.

[29] Ojha S., Dorling J., Battersby C., Longford N., Gale C. (2019). Optimising nutrition during therapeutic hypothermia. Arch. Dis. Child. Fetal Neonatal Ed..

[30] Biskop E., Paulsdotter T., Hellström Westas L., Ågren J., Blomqvist Y.T. (2019). Parental participation during therapeutic hypothermia for neonatal hypoxic-ischemic encephalopathy. Sex. Reprod. Healthc..

[31] Craig A., Deerwester K., Fox L., Jacobs J., Evans S. (2019). Maternal holding during therapeutic hypothermia for infants with neonatal encephalopathy is feasible. Acta Paediatr..

[32] Ranger M., Zwicker J.G., Chau C.M.Y., Park M.T.M., Chakravarthy M.M., Poskitt K., Miller S.P., Bjornson B.H., Tam E.W.Y., Chau V. (2015). Neonatal pain and infection relate to smaller cerebellum in very preterm children at school age. J. Pediatr..

[33] Chau C.M.Y., Ranger M., Bichin M., Park M.T.M., Amaral R.S.C., Chakravarthy M., Poskitt K., Synnes A.R., Miller S.P., Grunau R.E. (2019). Hippocampus, amygdala, and thalamus volumes in very preterm children at 8 years: Neonatal pain and genetic variation. Front. Behav. Neurosci..

[34] Zwicker J.G., Miller S.P., Grunau R.E., Chau V., Brant R., Studholme C., Liu M., Synnes A., Poskitt K.J., Stiver M.L. (2016). Smaller cerebellar growth and poorer neurodevelopmental outcomes in very preterm infants exposed to neonatal morphine. J. Pediatr..

[35] Anand K.J.S., Hall R.W., Desai N., Shepherd B., Bergqvist L.L., Young T.E., Boyle E.M., Carbajal R., Bhutani V.K., Moore M.B. (2004). Effects of morphine analgesia in ventilated preterm neonates: Primary outcomes from the NEOPAIN randomised trial. Lancet.

[36] Azzopardi D., Brocklehurst P., Edwards D., Halliday H., Levene M., Thoresen M., Whitelaw A., TOBY Study Group (2008). The TOBY Study. Whole body hypothermia for the treatment of perinatal asphyxial encephalopathy: A randomised controlled trial. BMC Pediatr..

[37] Eicher D.J., Wagner C.L., Katikaneni L.P., Hulsey T.C., Bass W.T., Kaufman D.A., Horgan M.J., Languani S., Bhatia J.J., Givelichan L.M. (2005). Moderate hypothermia in neonatal encephalopathy: Safety outcomes. Pediatr. Neurol..

[38] Shankaran S., Laptook A.R., Ehrenkranz R.A., Tyson J.E., McDonald S.A., Donovan E.F., Fanaroff A.A., Poole W.K., Wright L.L., Higgins R.D. (2005). Whole-body hypothermia for neonates with hypoxic-ischemic encephalopathy. N. Engl. J. Med..

[39] Jacobs S.E., Morley C.J., Inder T.E., Stewart M.J., Smith K.R., McNamara P.J., Wright I.M.R., Kirpalani H.M., Darlow B.A., Doyle L.W. (2011). Whole-body hypothermia for term and near-term newborns with hypoxic-ischemic encephalopathy: A randomized controlled trial. Arch. Pediatr. Adolesc. Med..

[40] Simbruner G., Mittal R.A., Rohlmann F., Muche R., neo.nEURO.network Trial Participants (2010). Systemic hypothermia after neonatal encephalopathy: Outcomes of neo.nEURO.network RCT. Pediatrics.

[41] Gunn A.J., Gunn T.R., de Haan H.H., Williams C.E., Gluckman P.D. (1997). Dramatic neuronal rescue with prolonged selective head cooling after ischemia in fetal lambs. J. Clin. Investig..

[42] Vutskits L., Xie Z. (2016). Lasting impact of general anaesthesia on the brain: Mechanisms and relevance. Nat. Rev. Neurosci..

[43] Sabir H., Dingley J., Scull-Brown E., Chakkarapani E., Thoresen M. (2018). Fentanyl induces cerebellar internal granular cell layer apoptosis in healthy newborn pigs. Front. Neurol..

[44] Montaldo P., Vakharia A., Ivain P., Mendoza J., Oliveira V., Markati T., Shankaran S., Thayyil S. (2020). Pre-emptive opioid sedation during therapeutic hypothermia. Arch. Dis. Child. Fetal Neonatal Ed..

[45] Mohammad K., McIntosh S., Lee K.S., Beltempo M., Afifi J., Tremblay S., Shah P., Wilson D., Bodani J., Khurshid F. (2023). Variations in care of neonates during therapeutic hypothermia: Call for care practice bundle implementation. Pediatr. Res..

[46] Berube M.W., Lemmon M.E., Pizoli C.E., Bidegain M., Tolia V.N., Cotton C.M., Greenberg R.G. (2020). Opioid and benzodiazepine use during therapeutic hypothermia in encephalopathic neonates. J. Perinatol..

[47] Natarajan G., Shankaran S., Laptook A.R., McDonald S.A., Pappas A., Hintz S.R., Das A., NICHD Neonatal Research Network (NRN) Whole Body Hypothermia Subcommittee (2018). Association between sedation-analgesia and neurodevelopment outcomes in neonatal hypoxic-ischemic encephalopathy. J. Perinatol..

[48] Liow N., Montaldo P., Lally P.J., Teiserskas J., Bassett P., Oliveira V., Mendoza J., Slater R., Shankaran S., Thayyil S. (2020). Preemptive morphine during therapeutic hypothermia after neonatal encephalopathy: A secondary analysis. Ther. Hypothermia Temp. Manag..

[49] Gundersen J.K., Chakkarapani E., Jary S., Menassa D.A., Scull-Broen E., Frymoyer A., Walloe L., Thoresen M. (2021). Morphine and fentanyl exposure during therapeutic hypothermia does not impair neurodevelopment. EClinicalMedicine.

[50] Lugli L., Spada C., Garetti E., Guidotti I., Roversi M.F., Della Casa E., Bedetti L., Lucaccioni L., Pugliese M., Ferrari F. (2022). Fentanyl analgesia in asphyxiated newborns treated with therapeutic hypothermia. J. Matern. Fetal Neonatal Med..

[51] Frymoyer A., Bonifacio S.L., Drover D.R., Su F., Wustoff C.J., Van Meurs K.P. (2017). Decreased morphine clearance in neonates with hypoxic ischemic encephalopathy receiving hypothermia. J. Clin. Pharmacol..

[52] Favié L.M.A., Groenendaal F., van den Broek M.P.H., Rademaker C.M.A., de Hann T.R., van Straaten H.L.M., Dijk P.H., van Heijst A., Dudink J., Dijkman K.P. (2019). Pharmacokinetics of morphine in encephalopathic neonates treated with therapeutic hypothermia. PLoS ONE.

[53] Lugli L., Garetti E., Goffredo B.M., Candia F., Crestani S., Spada C., Guidotti I., Bedetti L., Miselli F., Della Casa E.M. (2023). Continuous fentanyl infusion in newborns with hypoxic-ischemic encephalopathy treated with therapeutic hypothermia: Background, aims, and study protocol for time-concentration profiles. Biomedicines.

[54] Lewis S.R., Nicholson A., Smith A.F., Alderson P. (2015). Alpha-2 adrenergic agonists for the prevention of shivering following general anaesthesia. Cochrane Database Syst. Rev..

[55] Gauda E.B., Chavez-Valdez R., Northington F.J., Lee C.K.K., Rudek M.A., Guglieri-Lopez B., Ivaturi V. (2022). Clonidine for sedation in infants during therapeutic hypothermia with neonatal encephalopathy: Pilot study. J. Perinatol..

[56] Shukla U., Malhotra K., Prabhakar T. (2011). A comparative study of the effect of clonidine and tramadol on post-spinal anaesthesia shivering. Indian. J. Anaesth..

[57] O’Mara K., Weiss M.D. (2018). Dexmedetomidine for sedation of neonates with hie undergoing therapeutic hypothermia: A single-center experience. AJP Rep..

[58] Hoffman W.E., Cheng M.A., Thomas C., Baughman V.L., Albrecht R.F. (1991). Clonidine decreases plasma catecholamines and improves outcome from incomplete ischemia in the rat. Anesth. Analg..

[59] Hoffman W.E., Kochs E., Werner C., Thomas C., Albrecht R.F. (1991). Dexmedetomidine improves neurologic outcome from incomplete ischemia in the rat. Reversal by the alpha 2-adrenergic antagonist atipamezole. Anesthesiology.

[60] Hoffman W.E., Lingamneni P., Minshall R., Miletich D.J., Albrecht R.F. (1993). Brain alpha 2-adrenergic receptor binding during incomplete cerebral ischemia in the rat. Anesth. Analg..

[61] Yuan S.Z., Runold M., Hagberg H., Bona E., Lagercrantz H. (2001). Hypoxic-ischaemic brain damage in immature rats: Effects of adrenoceptor modulation. Eur. J. Paediatr. Neurol..

[62] Basker S., Singh G., Jacob R. (2009). Clonidine in paediatrics—A review. Indian. J. Anaesth..

[63] Agthe A.G., Kim G.R., Mathias K.B., Hendrix C.W., Chavez-Valdez R., Jansson L., Lewis T.R., Yaster M., Gauda E.B. (2009). Clonidine as an adjunct therapy to opioids for neonatal abstinence syndrome: A randomized, controlled trial. Pediatrics.

[64] Chen X., Chen D., Li Q., Wu S., Pan J., Liao Y., Zheng X., Zeng W. (2021). Dexmedetomidine alleviates hypoxia-induced synaptic loss and cognitive impairment via inhibition of microglial nox2 activation in the hippocampus of neonatal rats. Oxid. Med. Cell Longev..

[65] Laudenbach V., Mantz J., Lagercrantz H., Desmonts J.M., Evrard P., Gressens P. (2002). Effects of alpha(2)-adrenoceptor agonists on perinatal excitotoxic brain injury: Comparison of clonidine and dexmedetomidine. Anesthesiology.

[66] Ma D., Hossain M., Rajakumaraswamy N., Arshad M., Sanders R.D., Franks N.P., Maze M. (2004). Dexmedetomidine produces its neuroprotective effect via the alpha 2A-adrenoceptor subtype. Eur. J. Pharmacol..

[67] Paris A., Mantz J., Tonner P.H., Hein L., Brede M., Gressens P. (2006). The effects of dexmedetomidine on perinatal excitotoxic brain injury are mediated by the alpha2A-adrenoceptor subtype. Anesth. Analg..

[68] Jiang L., Hu M., Lu Y., Cao Y., Chang Y., Dai Z. (2017). The protective effects of dexmedetomidine on ischemic brain injury: A meta-analysis. J. Clin. Anesth..

[69] Chrysostomou C., Schulman S.R., Herrera Castellanos M., Cofer B.E., Mitra S., Garcia da Rocha M., Wisemandle W.A., Gramlich A. (2014). A phase II/III, multicenter, safety, efficacy, and pharmacokinetic study of dexmedetomidine in preterm and term neonates. J. Pediatr..

[70] Elliott M., Burnsed J., Heinan K., Letzkus L., Andris R., Fairchild K., Zanelli S. (2022). Effect of dexmedetomidine on heart rate in neonates with hypoxic ischemic encephalopathy undergoing therapeutic hypothermia. J. Neonatal Perinatal Med..

[71] Acun C., Ali M., Liu W., Karnati S., Fink K., Aly H. (2024). Effectiveness and safety of dexmedetomidine in neonates with hypoxic ischemic encephalopathy undergoing therapeutic hypothermia. J. Pediatr. Pharmacol. Ther..

[72] Cosnahan A.S., Angert R.M., Jano E., Wachtel E.V. (2021). Dexmedetomidine versus intermittent morphine for sedation of neonates with encephalopathy undergoing therapeutic hypothermia. J. Perinatol..

[73] Naveed M., Bondi D.S., Shah P.A. (2022). Dexmedetomidine versus fentanyl for neonates with hypoxic ischemic encephalopathy undergoing therapeutic hypothermia. J. Pediatr. Pharmacol. Ther..

[74] Surkov D. (2019). Is dexmedetomidine a potential neuroprotective agent for term neonates with hypoxic-ischemic encephalopathy?. Pediatr. Anesth. Crit. Care J.—PACCJ.

[75] McAdams R.M., Pak D., Lalovic B., Phillips B., Shen D.D. (2020). Dexmedetomidine pharmacokinetics in neonates with hypoxic-ischemic encephalopathy receiving hypothermia. Anesthesiol. Res. Pract..

[76] Baserga M., DuPont T.L., Ostrander B., Minton S., Sheffield M., Balch A.H., Timothy T.M., Watt K.M. (2021). Dexmedetomidine use in infants undergoing cooling due to neonatal encephalopathy (Dice trial): A randomized controlled trial: Background, aims and study protocol. Front. Pain Res..

[77] van den Broek M.P.H., van Straaten H.L.M., Huitema A.D.R., Egberts T., Toet M.C., de Vries L.S., Rademaker K., Groenendaal F. (2015). Anticonvulsant effectiveness and hemodynamic safety of midazolam in full-term infants treated with hypothermia. Neonatology.

[78] Liu Y., Barks J.D., Xu G., Silverstein F.S. (2004). Topiramate extends the therapeutic window for hypothermia-mediated neuroprotection after stroke in neonatal rats. Stroke.

[79] Barks J.D., Liu Y.Q., Shangguan Y., Silverstein F.S. (2010). Phenobarbital augments hypothermic neuroprotection. Pediatr. Res..

[80] Bittigau P., Sifringer M., Genz K., Reith E., Pospischil D., Govindarajalu S., Dzietko M., Pesditschek S., Mai I., Dikranian K. (2002). Antiepileptic drugs and apoptotic neurodegeneration in the developing brain. Proc. Natl. Acad. Sci. USA.

